# Stem cell therapy in systemic sclerosis

**DOI:** 10.1007/s10067-025-07557-y

**Published:** 2025-07-02

**Authors:** Lazaros I. Sakkas, Theodora Simopoulou, Ioannis Alexiou, Christos Liaskos, Ian C. Chikanza

**Affiliations:** 1 Faculty of Medicine, School of Health Sciences, University of Thessaly, 41110 Larissa, Greece; 2 Department of Rheumatology and clinical Immunology, Faculty of Medicine, School of Health Sciences, University of Thessaly, Larissa, 41 110 Greece; 3 Department of Rheumatology and clinical Immunology, University General Hospital of Larissa, Larissa, 41 110 Greece; 4 Internal Medicine clinic, IASO General Hospital, Larissa, 41 500 Greece; 5 Pediatrics Department, University of Zimbabwe, Harare, Zimbabwe; 6 International Arthritis and Hypermobility Centre, Harley Street Clinic, London, UK

**Keywords:** Adipose-derived mesenchymal stem cells, Bone marrow-derived mesenchymal stem cells, Haematopoietic stem cells, Mesenchymal stem cells, Stem cell transplantation

## Abstract

Systemic sclerosis is a complex autoimmune disease with widespread fibrosis in skin and internal organs, microvasculopathy, and autoantibodies. The disease causes ischemic changes and leads to impairment of internal organs with reduced quality of life and life expectancy. The pathogenesis is not clearly known but involves adaptive and innate immune cells which infiltrate skin lesions mostly early in the disease process. Current treatment is based on immunosuppressives, but there is a significant unmet therapeutic need, and a new therapeutic approach is required. Autologous haematopoietic stem cell transplantation appears to be an effective therapeutic option for SSc but requires standardization to reduce transplant-related mortality and post-transplant adverse effects. Mesenchymal stem cells (MSCs), exerting immunosuppressive, antifibrotic, and angiogenic actions, appear to be a promising therapeutic option but require further refinement. MSC-derived microvesicles retain MSC functions and circumvent some of the MSC challenges and thus may provide a more favorable therapeutic approach.

**Table:** 

Key Points • *There are unmet therapeutic needs for systemic sclerosis.* • *Autologous haematopoietic stem cell transplantation is an effective therapeutic option but needs standarization.* • *Mesenchymal stem cells are a promising therapeutic option but requires refinement.*

Key Points

• *There are unmet therapeutic needs for systemic sclerosis.*

• *Autologous haematopoietic stem cell transplantation is an effective therapeutic option but needs standarization.*

• *Mesenchymal stem cells are a promising therapeutic option but requires refinement.*

Systemic sclerosis (SSc) is a complex disease with widespread fibrosis, microvasculopathy, and immune cell activation. Microvasculopathy is expressed as an exaggerated vasospastic response to cold and fibro-intimal proliferation causing ischemia. Immune cell activation is evident by the presence of many autoantibodies (autoAbs), some of which are disease-specific, such as anti-DNA topoisomerase I (ATA, formerly anti-Scl70), anticentromere (ACA), and anti RNA polymerase III (ARPA) autoAbs. These pathophysiological changes cause skin fibrosis, digital ulcers, and internal organ impairment, leading to poor disease-related quality of life and increased mortality [1].

The pathogenesis of the disease is incompletely known. The involvement of microvessels and the activation of the immune system involving T cells, B cells, and innate immune cells occurs very early before overt fibrosis [2–4]. Immune cell signatures in SSc skin are present in early disease and subsequently slowly disappear in long-lasting disease [5]. Currently, immunosuppressives form the basis of therapy for SSc, and lately, an antifibrotic agent has been added, particularly for SSc-associated interstitial lung disease (SSc-ILD) [6, 7]. However, the efficacy of immunosuppressives is modest, and there is an urgent need for a different therapeutic approach. A cellular therapy with autologous chimeric antigen receptor (CAR)-T cells has been recently tested in a few SSc patients with encouraging results, whereas stem cell transplantation offers another therapeutic strategy for this frequently devastating disease.

## Stem cell therapy

Stem cells are pluripotent cells capable of differentiating into different lineages. There are two types of stem cells with therapeutic interest for autoimmune diseases: haematopoietic stem cells (HSCs) and mesenchymal stem cells (MSCs).

### Autologous haematopoietic stem cells

The rationale behind the HSC transplantation (HSCT) is to delete autoreactive immune cells and replace them with naive and more tolerant immune cells. HSCs currently used for SSc are autologous (AHSCs), since allogeneic HSCs are highly immunogenic, causing graft-versus-host disease (GVHD). AHSCs are collected from peripheral blood after leukapheresis, but the procedure thus far has not been standardized. It involves mobilization of stem cells with cyclophosphamide (CyP) and granulocyte colony-stimulating factor (GCSF), and AHSCs can be CD34-selective to enrich for HSCs or unmanipulated. A conditioning regimen, used to delete autoreactive immune cells, ranges in intensity from non-myeloablative to myeloablative. The latter usually involves total body irradiation and high-dose chemotherapy plus/minus anti-thymocyte globulin [8–10].

Autologous HSCT (AHSCT) causes bone marrow aplasia, and neutrophils and platelets reconstitution occur within 13 days after graft infusion, monocytes, natural killer cells, and dendritic cells within a month, while T cells and B cells reconstitution occurs within 6 months [8, 11, 12]. In a CD34-selective AHSCT, with high-dose CyP as a sole conditioning regimen, CD8 + cells recovered within a month, but CD4+T cells remained low for more than 36 months [13].

**Fig. 1:**
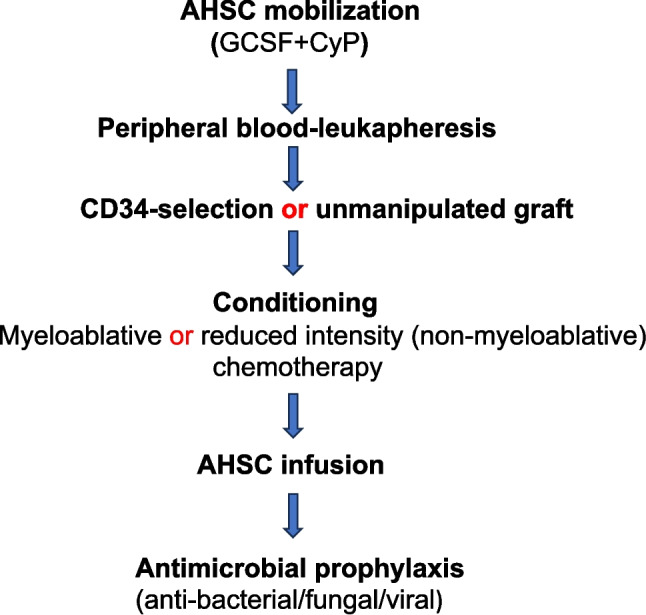
Flowchart of autologous haematopoietic stem cell(AHSC) transplantation. CyP: cyclophosphamide; GCSF: granulocyte colony stimulating factor

During this vulnerable period, patients receive antibacterial/antifungal prophylaxis and antiviral prophylaxis for a year. Related to the post-AHSCT period is a condition termed engraftment syndrome that occurs in up to 40% of patients and encompasses a constellation of features, including fever, liver dysfunction, renal dysfunction, and capillary leakage [14]. A flowchart of the AHSCT transplantation is shown in Fig. 1.

### Mesenchymal stem cells

Mesenchymal stem cells (MSCs) are a heterogeneous population of stromal cells with differentiation potential towards mesenchymal tissue cells, such as muscle cells, osteoblasts, chondrocytes, and adipocytes [15]. These cells adhere to plastic, expand easily in culture, and exhibit surface expression of stromal markers CD73(L-VAP-2), CD90(Thy-1), CD13(APN), and CD105(endoglin), but no surface expression of haematopoietic lineage markers, such as CD34, CD19, CD45 (common leukocyte antigen), CD14, and HLA-DR [16, 17]. MSCs can derive from the vascularized stroma of different sources, including bone marrow (BM), adipose tissue (AD), tooth pulp, umbilical cord (UC) (Whearton’s jelly), and placenta [18]. However, there may be differences among MSCs from different sources. The International Federation for Adipose Therapeutics and Science (IFATS) and the International Society for Cellular Therapy (ISCT) issued a statement for the identification of adipose-derived uncultured stromal vascular fraction (SVF) and the adherent stromal/mesenchymal cells (ASCs) (CD34 ± CD45 − CD90 + CD73 + CD13 + CD105 + CD10 + CD36 + CD31 −). Adipose tissue–derived SVF (AD-SVF) contains 2–10% MSCs [17].

MSCs are expanded in culture and usually primed (preconditioned), i.e., stimulated with various agents in culture, to modulate their functions. MSCs exert immunosuppressive functions on T cells and macrophages by suppressing T cell proliferation and production of transforming growth factor (TGF)β, and by inhibition of B cell differentiation and antibody production, and natural killer (NK) cell proliferation [19–22]. Human bone marrow (BM)-MSCs inhibit T cell proliferation and chemokine production, as well as adhesion molecules involved in trans-endothelial migration [23].

It should be noted that MSCs are not immunosuppressives constitutively but acquire different functions depending on the priming [24]. MSC secretome generally depends on cell source, purity of cells, and preconditioning, and this has also been shown for MSC angiogenic properties [25]. Interferon (IFN)γ is usually used to prime MSCs to upregulate indoleamine 2,3 dioxygenase (IDO), crucial for their immunosuppression. Also, adipose-derived MSCs from mice preconditioned with low-molecular weight heparin exhibited higher anti-inflammatory and antifibrotic effects on a bleomycin (BLM)-induced fibrosis mouse model [26]. Depending on priming, human adipose tissue–derived MSCs (AD-MSCs) can increase the production of haematopoietic growth factors (hepatocyte growth factor, granulocyte/monocyte colony–stimulating factor), and proinflammatory cytokines (IL-6, IL-8) supporting hematopoiesis and angiogenesis [27]. Therefore, relevant studies should be interpreted with caution, as they include variations in cell source, cell purity, and priming. MSCs exhibit low immunogenicity since they express low levels of HLA class I and HLA class II [28, 29]. Nevertheless, allogeneic MSCs are recognized and lysed by NK cells [20, 21, 30], whereas allogeneic bone marrow–derived MSCs (BM-MSCs) stimulated graft rejection in a non-myeloablative setting [30].

MSCs from SSc patients may differ from those of healthy donors. BM-MSCs from SSc patients did not differ from those from healthy donors in immunosuppressive effect on T cell proliferation and differentiation potential [31, 32]. They increased interleukin (IL)−6 and TGFβ production and induced regulatory T cells (Tregs) [32] but also exhibited exaggerated differentiation to myofibroblasts [33]. Other studies reported that BM-MSCs from SSc patients display the same phenotype as healthy controls but exhibit impaired endothelial cell differentiation [34], although BM-MSCs from early SSc patients can stimulate angiogenesis in vitro [35].

AD-MSCs from SSc patients showed equivalent differentiation capacity, immunosuppressive or proangiogenic properties as those from healthy donors, although their proliferation rate and metabolic activity were reduced [36, 37]. Others reported that AD-MSCs from SSc or SLE patients were less effective in suppressing autologous T cell activation [38]. It should be emphasized that the SSc microenvironment promotes fibrosis. For instance, AD-MSCs from healthy donors exposed to dermal blister fluid from diffuse cutaneous SSc (dcSSc) patients developed a myofibroblast signature and enhanced osteogenic commitment [39]. Other studies clearly showed that AD-MSCs from SSc patients exhibit pathogenic properties. BM-MSCs and AD-MSCs from SSc patients exhibited profibrotic microRNA profiling [40], promoted profibrotic Th2 differentiation with decreased IFNγ/IL-4 ratio, and upregulated T helper (Th)17 and Tregs [38]. However, AD-SVF from SSc patients retained near-normal angiogenic potential [41].

Overall, MSCs may provide a favorable therapeutic option for SSc, since they have antifibrotic, immunosuppressive, and proangiogenic properties [42]. A summary of differences between HSCs and MSCs is shown in Table 1.

**Table 1:** Differences between haematopoietic stem cells (HSCs) and mesenchymal stem cells (MSCs)

Item	HSCs (Ref)	MSCs (Ref)
Source	PB-leukapheresis after mobilization with CyP and GCSF	Vascularized stroma from various tissues (adipose tissue, bone marrow, tooth pulp, umbilical cord, placenta) [18]
Lineage	Haematopoietic	Heterogeneous population with stromal cell markers but no haematopoietic lineage markers [15]
Selection	CD34-selective or unmanipulated	They selected by adherence to plastic, expansion in culture, and use of many cell surface markers [16–18]
Purity of cells	High/medium	Low/medium. It requires the use of many cell surface markers [16, 17]
Priming	No	Yes. It is required to modulate their function which depends on cell source, purity of cells, and priming [19–22, 24–30]
Immunogenicity	High	Low [20, 21, 28–30]

CyP: cyclophosphamde; GCSF: granulocyte colony-stimulating factor; PB: peripheral blood

## Autologous haematopoietic stem cell transplantation in systemic sclerosis

AHSCT has been used for refractory SSc cases. After the first reports and phase I/II clinical trials with encouraging results [43–49], three randomized controlled trials (RCTs) were conducted in early severe SSc and compared AHSCT with IV pulses of CyP: the ASSIST trial, an unmanipulated, non-myeloablative AHSCT [8], the ASTIS trial, a CD34-selective, non-myeloablative AHSCT [9], and the SCOT trial, a CD34-selective, myeloablative AHSCT [10]. At 4 years post-transplant, progression/event-free survival (P/EFS) was reported in 81% of the AHSCT group versus 74% in the CyP group in the ASTIS trial, and 79% versus 50%, respectively, in the SCOT trial. At 6 years, the respective figures for EFS were 74% versus 61% in the ASTIS trial, and 74% versus 47% in the SCOT trial [9, 10]. Overall survival was also greater in AHSCT compared to the CyP group. At 4 years, survival was 83.5% in the AHSCT group versus 74% in the CyP group in the ASTIS trial, and at 6 years survival was 86% in the AHSCT group versus 51% in the CyP group in the SCOT trial [9, 10]. AHSCT led to great improvement in skin thickness, as assessed by modified Rodnan skin score (mRSS), functional ability, health-related quality of life (HRQoL), and forced vital capacity (FVC) [9, 10]. An unmanipulated, non-myeloablative AHSCT improved mRSS and FVC at 1, 2, and 3 years, but not diffusing lung capacity for carbon monoxide (DLCO), and at 5-year survival, it was 78% and PFS was 70% [50]. A recent systematic review and meta-analysis of 3 RCTs and 19 observational studies showed that AHSCT improved skin score and lung function and resulted in high survival rates at 2 years post-transplant favoring AHSCT [51]. In a more recent single-arm prospective study of 20 patients with high-risk dcSSc, non-selective, non-myeloablative AHSCT, followed by mycophenolate mofetil (MMF) maintenance, resulted in overall survival and event-free survival at 5 years of 85% and 75%, respectively [12]. A retrospective study from the German Registry of 80 patients with dcSSc who underwent AHSCT reported higher overall survival for AHSCT patients at 15 years compared to controls (92% vs 71% of anti-Scl70 + men controls) [52]. It should be noted that small doses of CyP plus Thiotera were used as conditioning chemotherapy in patients with cardiac involvement in this study [52]. Neutrophil to lymphocyte ratio was decreased in AHSCT responsive patients and correlated with mRSS and FVC [53]. ILD ground glass opacities on high-resolution computed tomography (HRCT) were significantly decreased at 12 months and 24 months after AHSCT [54]. By handling longitudinal missing data, a study found that patients with the inflammatory and fibroproliferative SSc subset had superior long-term improvement in lung function, both FVC and DLCO, and quality of life compared to CyP [55]. In a small study, CD34-selective AHSCT resulted in progressive improvement of skin score for 3 years whereas ATA, Krebs von den Lungen-6 (KL-6), and surfactant protein D, markers of ILD, progressively decreased [13]. AHSCT stabilized or normalized esophageal peristalsis but had no effect on unmeasurable peristalsis [56].

SSc reactivation after AHSCT was reported in 22% of AHSCT patients by 24 months in the ASTIS trial, but only in 9% of patients in the SCOT trial and greatly favored AHSCT over CyP. Generally, SSc progression occurred in 20% of patients after AHSCT over 5–7 years [9, 10, 50, 57], and rituximab (RTX) was an efficacious treatment option for these patients [58].

The rationale underlying AHSCT was to eliminate autoreactive T and B cells and replace them with a more normal immune system, i.e., to reset the immune system. Indeed, sequence analysis of immunoglobulin heavy chain genes from patients in the SCOT trial revealed a resetting of B cells to a naive status in the AHSCT group but not in the CyP group [59]. Similarly, after AHSCT, T cell receptor (TCR) diversity increased [60], and in SLE patients, remission after AHSCT was associated with normalization of TCR repertoire, disappearance of anti-dsDNA Abs, and restoration of Tregs [61]. Also, the suppressive capacity of regulatory B cells was restored after unselective AHSCT [62]. In a CD34-selective AHSCT, with CyP as a sole conditioning regimen, the majority of T cells post-transplant were antifibrotic Th1 cells [13]. A molecular analysis of the SCOT trial patients at baseline, 6 months, and 26 months post-transplant showed improved IFN, NK, and neutrophil molecular signatures in post-AHSC patients, but not in CyP-treated patients [63].

However, treatment-related mortality (TRM) remains high in AHSCT. In early studies, TRM was very high at 16% (RR 9.0) (95% CI 1.57–51.7) [46, 64]. A CD34-selective, non-myeloablative AHSCT in the ASTIS trial had a high TRM at 10.1% during the first year, whereas a CD34-selective myeloablative AHSCT in the SCOT trial had a low TRM at 3% at 4 years. This latter low figure was perhaps due to selection of patients (excluding patients with severe lung or heart impairment). In an unselected, non-myeloablative AHSCT, TRM was 6% [50], and in a recent systematic review and meta-analysis, a pooled TRM was 6.3% (95%CI 4.2–8.4) [51]. A subsequent single-center study found that high-dose chemotherapy AHSCT was associated with very high TRM at 18.8% [65], whereas a single-arm prospective study of 20 patients with high-risk dcSSc, non-selective, non-myeloablative AHSCT, followed by MMF maintenance, resulted in TRM of 10% at 1 year [12].

Patients for AHSCT should be carefully selected, as severe cardiopulmonary impairment has been identified as a risk factor for early TRM [66, 67]. In SSc-myocarditis, CyP-related cardiotoxicity was avoided in one patient by a two-step approach with a combination of RTX plus MMF followed by a small conditioning CyP dose [68]. In a large retrospective study, small doses of CyP plus thiotera, as a conditioning regimen, in patients with cardiac involvement were very gratifying, resulting in a very high overall survival at 15 years [52]. Baseline low estimated glomerular filtration rate (eGFR) was also a risk factor for TRM [12].

Severe infections and particularly viral infections were common after AHSCT, and late varicella-zoster virus (VZV) was diagnosed in 36% of patients in the SCOT trial, whereas cancer was also of concern [10]. Thrombotic microangiopathy and secondary autoimmune disease have also been reported [14, 69].

There were various systematic reviews and meta-analyses to decipher the complexity of the procedure and find optimal parameters for a better patient outcome. An analysis of the three RCTs found a better overall survival for non-myeloablative CD34-selective ASCT at 10 years and for myeloablative CD34-selective AHSCT at 6 years over CyP [70]. AHSCT of all types improved skin thickness relative to CyP, and functional ability was increased in the non-myeloablative CD34-selective and myeloablative CD34-selective AHSCT [70].

It should be noted that CD34-selection is used to enrich HSCs and remove autoreactive T cells and B cells from the graft. In 2016, an analysis of the European Society for Blood and Marrow Transplantation (EBMT) Autoimmune Diseases Working Party for the effect of CD34-selection of AHSCT on the outcome of 138 patients with SSc showed that CD34-graft selection did not add any benefit on overall survival or incidence of disease progression [71]. However, a later analysis showed that CD34+selective were more effective than unmanipulated AHSCs [72]. In a phase I/II Japanese trial, comparing selective vs unmanipulated AHSCT, with CyP as a sole conditioning regimen, progression-free survival was 81.8% in the CD34-selective group vs 50% in the unmanipulated group [72]. No TRM was reported in either group [47, 72]. Moreover, in the CD34-selective group, skin improvement was greater, and FVC continuously increased over 8 years, whereas in the unmanipulated group, FVC returned to baseline at 3 years [72]. A recent study reported a 2-year progression-free survival of 81% after AHSCT and better outcome for CD34-selection [73]. Toxicity and viral infections (Cytomegalovirus [CMV], VZV) were more frequent in the CD34-selective group [72]. In a review, both non-myeloablative CD34-selective and myeloablative CD34-selective AHSCT improved event-free survival but had more serious adverse events compared to CyP [70]. Mobilization of CD34-selection graft with reduced dose of CyP (2 × 1–1.5 g/m^2^) vs high CyP dose (2×2 g/m^2^) resulted in no difference in HSCs yield [74].

Allogeneic bone marrow transplantation has been tried in two patients with refractory SSc [75], but it was associated with fatal opportunistic infections and a high risk for GVHD [75]. TRM in allogeneic HSCT for autoimmune diseases was very high at around 20% and related to infection and GVHD [76].

Guidelines for the AHSCT in SSc have been published in 2017 [66]. Best candidates for AHSCT are patients within the first 4 years of rapidly progressive disease and a life-threatening condition with a risk 3–5 for survival [77], whereas exclusion criteria were PAH, LVEF < 45%, constrictive pericarditis, cardiac tamponade, severe arrhythmias, and severe lung disease (FVC < 45%) [66]. However, questions remain. For instance, the optimal conditioning regimen is to be defined. Also, the exact timing for AHSCT is not clear, i.e., upfront treatment for dcSSc or rescue treatment for refractory disease. At present, data from a large single-center cohort showed that patients eligible for, but excluded from AHSCT, had poor outcomes with survival 96% at 2 years, 77% at 5 years, and 75% at 10 years [78]. A new study with AHSCT as upfront treatment for dcSSc is underway and will answer this question [79].

Although CD34-selective, non-myeloablative AHSCT was more effective than RTX or conventional immunosuppressives [80], a recent Australian study reported that dcSSc patients meeting the ASTIS and SCOT inclusion criteria for AHSCT but not receiving AHSCT had similar EFS at 4 years, as patients who received AHSCT [81]. Also, a combination of MMF and RTX compared to AHSCT in patients eligible for AHSCT had similar effects on skin and ILD improvement at 24 months but a better safety profile [82].

## Mesenchymal stem cell transplantation in systemic sclerosis

### Transplantation in experimental fibrosis

In bleomycin (BLM)-induced SSc mouse model [83–85], and in hypochlorite (HOCl)-induced SSc model, [86–88], BM-MSCs or umbilical cord–derived MSCs (UC-MSCs) decreased skin and lung fibrosis, autoantibody levels, and proinflammatory cytokines. They also increased antioxidant capacity [86]. Human UC-MSCs also improved skin fibrosis and decreased Th17 skin infiltration [85] and restored gut microbiota composition and function in BLM-induced SSc mice [89]. Also, AD-MSCs attenuated skin and lung fibrosis and decreased Th2 cells and B cells in the spleen and T cell and macrophage infiltration and IL-13 and IL-6 cytokines in the skin of BLM-induced SSc mice [90]. In HOCl-induced fibrosis BALB/c mice, BM-MSCs from syngeneic, allogeneic, and xenogeneic (human) donors decreased skin and lung fibrosis, and this effect was not associated with MSC migration to the skin or with long-term survival of MSCs [91] whereas inducible nitric oxide synthase (iNOS) was required for the anti-fibrotic effect of BM-MSCs [92]. In a sclerodermatous GVHD model, BM-MSCs were not detected in the skin but decreased skin fibrosis by inhibiting the migration of T cells and macrophages to the skin [93]. Finally, in the DNA-topoisomerase I-induced SSc mouse model, allogeneic and syngeneic BM-MSCs similarly reduced skin and lung inflammation and fibrosis [94].

### Transplantation in human systemic sclerosis

#### Local graft delivery

Local injection of adipose tissue–derived stromal cells has been tested mostly for ischemic acral lesions in SSc patients. In one study, autologous adipose–derived stem cells in hyaluronic acid were injected locally at the sclerotic areas in 6 patients with localized scleroderma (one with generalized morphea and 5 with linear scleroderma) and improved skin tightness [95].

In 15 SSc patients with long-standing digital ulcers (DUs), autologous adipose–derived cell fractions were injected subcutaneously at the base of the corresponding fingers and led to rapid healing of DUs [96]. Moreover, capillaries on nailfold video capillaroscopy (NVC) significantly increased [96]. The same group conducted a RCT with adipose tissue cells injected at the base of fingers of SSc patients with DUs and showed healing of DUs in 23 of 25 patients (vs 1/13 controls) [97]. In a pilot study, BM-derived mononuclear cells containing endothelial progenitor cells were injected into ischemic limbs of patients with DUs and led to improvement, but at 2 years, there was a relapse in 18% of patients that led to limb amputation in 9% of patients [98]. In another study of 8 patients with refractory DUs, BM-derived mononuclear cells injected intramuscularly into ischemic limbs decreased pain and diminished DUs, and these effects were associated with the improvement of blood flow and new capillaries in nailbeds [99]. Local delivery of BM-derived mononuclear cells containing endothelial progenitor cells appears to be a gratifying approach for ischemic acral lesions, as it resulted in an amputation-free rate of 97.4% at 10 years [100].

Autologous adipose-derived stromal vascular fraction (AD-SVF) was used in an open-label, single-arm study of 12 SSc patients with hand disability [100]. AD-SVF was injected subcutaneously at the base of each finger and improved hand function, severity of Raynaud’s phenomenon, DU number, and quality of life [101], which were maintained at 12 months and 24 months of follow-up [102]. However, mRSS, focused on hands, did not decrease significantly [102]. Importantly, there were no severe adverse effects [101]. AD-SVF is a heterogeneous population of cells with MSCs, endothelial progenitor cells, pericytes, hematopoietic cells, and immune cells, and autologous AD-SVF from SSc patients retains near-normal angiogenic capacity [41].

In a proof-of-concept study, autologous AD-SVF, injected into multiple sites of each finger of SSc patients with hand disability, improved skin fibrosis, pain, and quality of life and led to healing of 31.6% of active DUs at 24 weeks [103]. Also, in a randomized open-label trial, autologous AD-SVF, injected at multiple sites of the hands of SSc patients, improved pain and DU number [104]. In an RCT, Celution-processed autologous adipose-derived regenerative cells from SSc patients, injected into fingers, led to marginal improvement of hand function [105]. The Celution system is an automated processing of adipose-derived regenerative cells in a closed system [106]. Similarly, in another double-blind RCT of 40 SSc patients with hand dysfunction, autologous AD-SVF, processed with Celution 800/CRS and injected into fingers, was not superior to placebo [107].

#### Systemic graft delivery

Most studies evaluated the effect of allogeneic MSCs in SSc. In a patient with critical limb ischemia, three IV pulses of autologous MSCs expanded in culture, reduced skin necrosis, and revascularized extremities, as detected by angiography [108]. Furthermore, skin immunohistochemistry revealed cell clusters with tube-like structures suggesting that autologous MSC transplantation may restore the vascular network in SSc [108].

In an early study with 5 patients with severe progressive SSc, allogeneic BM-MSCs improved skin score in 3 out of 4 patients, although temporarily in one patient [109, 110]. DUs and acral necrosis diminished/healed in 3 out of 3 patients but recurred in 2 patients at 6 to 12 months post-transplant. Serum creatine phosphokinase (CPK) levels were normalized in one of 2 patients with myositis, DLCO increased in one of 3 patients, and pericardium calcification was worsened in one patient with this manifestation. ANA titers did not change [109, 110]. In a proof-of-concept phase 1/2 open-label trial in 20 patients with severe refractory SSc, a single IV infusion of allogeneic BM-MSCs improved mRSS, stabilized FVC and DLCO at 12 months, and resulted in progression-free survival in 75% of patients [111]. The procedure was safe with mild adverse effects in three patients [111]. There was an increase of IL-10-producing regulatory B cells (Bregs), especially in responders after BM-MSC infusion, suggesting that Bregs may be effector cells in allogeneic BM-MSC transplantation [112].

In a systematic review and meta-analysis of 9 studies with 133 SSc patients, MSC transplantation improved skin thickness, lung function (FVC and DLCO), DUs, and hand pain, and it was a relatively safe procedure with few adverse effects [113]. There was one death within a month post-transplant [114]. In a long follow-up of patients with autoimmune diseases, mostly SSc and SLE, allogeneic MSC infusion was a safe therapy, with TRM of 0.2% [115]. In a recent study, UC-MSC transplantation in 41 patients with SSc decreased mRSS from 18.7 to 14.0, to 13.3, and to 12.4, at 1, 3, and 5 years, respectively, whereas pulmonary arterial hypertension (PAH) remained stable in 5 of 8 patients. The overall survival at 5 years was 92.7%, and no adverse effect was reported [116]. In another recent report, BM-MSC or UC-MSC from healthy donors were transplanted into 113 patients with SSc and significantly improved 10-year survival [117]. A RCT protocol for BM-MSC transplantation in 20 SSc patients with refractory DUs was published [118].

Allogeneic MSC transplantation was also used in combination with plasmapheresis (PE). Allogeneic UC-MSC transplantation after 4 sessions of PE and subsequent RTX in 2 SSc patients with PAH and ILD led to marked clinical improvement and normalization of pulmonary artery pressure by echocardiography [119]. Upon recurrence of symptoms, allogeneic MSC alone stabilized disease [119]. Similarly, allogeneic UC-MSC transplantation after 3 PE sessions and subsequent CyP in 14 SSc patients improved skin thickness (mRSS from 20.1 to 13.8) at 12 months and significantly improved FVC, DLCO, and CT lung imaging in 3 patients with ILD [120]. This combination also improved hand function and normalized pulmonary arterial pressure in one patient with PAH [119, 120]. Serum ATA, TGFβ, and vascular endothelial growth factor (VEGF) levels decreased [120]. A summary of differences between AHSC transplantation and MSC transplantation is shown in Table 2.

**Table 2:** Comparison of autologous haematopoietic stem cell transplantation (HSCT) and systemic mesenchymal stem cell transplantation (MSCT) in SSc (ref)

Item	AHSCT(Reference)	MSCT(Reference)
Source	autologous peripheral blood after leukapheresis [8–10]	Allogeneic from tissues. Autologous MSCT may promote disease aggravation [31–35, 38, 40]
Conditioning	Yes (myeloablative or no-myeloablative) [8–10]	No
Priming of cells	No [8–10]	Yes. It is a requirement for MSC function [19–22, 24–27]
Immunogenicity	No [8–10]	Low. Low risk of rejection [20, 21, 28–30]
Effect on immune cells	It resets the immune system (more naive immune cells) [13, 59–63]	It increases Bregs [112] It downregulates TGFβ/Smad3 axis [128]
Efficacy	Efficacious. More effective than conventional immunosuppressives [8–10, 12, 13, 50–56, 70–73, 80]	Efficacy, but more data are needed [108–113, 116, 117, 119, 120] It improves vasculopathy [108]
Adverse effects	Bone marrow aplasia with high risk of infections and treatment-related mortality [8–13, 46, 50, 51, 64, 65, 70, 72] It requires anti-bacterial/fungal/viral prophylaxis. Engraftment syndrome [14] Secondary autoimmune disease [14, 69]	Minor and acceptable adverse effects [111–115]
Contraindication	severe cardiopulmonary disease [12, 52, 66–68]	No

In 2024, the EBMT published an expert-based position statement and clinical recommendations for cellular therapies in autoimmune diseases [121]. Cellular therapy should be considered in patients with progressive disease and active organ involvement that confers risk of organ failure (Table 3). Cellular therapy must be considered in multidisciplinary team meetings with external second opinions by hematologists, plus experts in autoimmune diseases, and approved by the ethics committee. Patients should be referred to accredited centers with multidisciplinary teams with hematologists and experts in autoimmune diseases.

**Table 3:** Cellular therapy for SSc (EBMT recommendations)(adapted from ref 121**)**

Age ≥ 18 years
Disease duration ≤ 5 years
Plus
1.mRSS>20 and ESR>25 and/or Hb<11g/dL
OR mRSS >15 and ≥ 1 major organ involvement
a. lung: DLCO and/or FFC<80% and ILD on CT scan. FVC < 45% is a contraindication for AHSCT
b: kidneys: past renal crisis OR stage 2/3 chronic renal failure (CrCl 30-89 ml/min).
c. heart: reversible heart failure OR rythm disturbances OR mild/moderate pericardial effusion. LVEF< 40%, constrictive pericarditis, cardiac tamponade, severe arrythmias, and PAH are contraindications for AHSCT.
2. Inadequate response to at least 2 immunosuppressives/antifibrotic medications currently used for SSc-ILD for a minimum 3 months
3. Referal to an expert accredited center with multidisciplinary team having haematologists and experts in SSc

AHSCT: autologous haematopoetic stem cell transplantation; DLCO:diffusion capacity for carbon monoxide; FVC:forced vital capacity; LVEF:left ventricular ejection fraction; mRSS: modified Rodnan skin score; CrCl:creatinine clearance; ILD:interstitial lung diseasePAH: pulmonary arterial hypertension

## Conclusion

Cellular therapies offer the opportunity for treatment-free disease remission in SSc patients refractory to current conventional treatments. AHSCT appears to generate a more naive immune system, improving skin thickness, quality of life, and survival, and is currently in the therapeutic armamentarium for SSc [122]. However, questions remain regarding timing, patient eligibility, conditioning regimen, and CD34 graft selection or not. Some researchers, but not all, found a better progression-free survival for CD34-selection [71–73]. Center experience and interdisciplinary collaboration are of paramount importance for the optimal outcome. However, other options are on the table for these patients. For instance, a combination of MMF with RTX compared with AHSCT in SSc patients eligible for AHSCT showed similar skin and lung efficacy. Moreover, at 24 months, event-free survival and safety profile favored the combination treatment [82].

MSCs appear to be a promising therapeutic option for SSc. However, challenges remain regarding MSC sourcing; characterization of cells; and standardization, efficacy, and rejection issues. Some of these issues, such as silencing β2m to inhibit HLA-class I to minimize graft rejection, and others to increase MSC efficacy, are being developed [123], as minor MHC antigen mismatched bone marrow–derived MSCs induced fibrosis in a scleroderma mouse model [124]. A novel approach was the generation of induced pluripotent cell lines from AD-MSCs from SSc patients, which differentiated into endothelial cells and thus can be of value for ischemic lesions in SSc [125]. MSC-derived microvesicles retain MSC functions and circumvent some of the problems facing MSCs and offer advantages over MSCs as a therapeutic option for systemic sclerosis [126, 127]. For instance, exosomes from AD-MSCs and AD-MSCs similarly alleviated skin fibrosis in BLM-induced SSc mice and inhibited collagen synthesis in skin fibroblasts from SSc patients by inhibiting TGFβ/Smad3 axis [128].

In conclusion, we need more data for the optimal procedure for AHSCT and for the standardization and efficacy of allogeneic MSCs for SSc in large RCTs.
